# End organ perfusion and pediatric microcirculation assessment

**DOI:** 10.3389/fped.2023.1123405

**Published:** 2023-09-29

**Authors:** Grace M. Arteaga, Sheri Crow

**Affiliations:** Department of Pediatric and Adolescent Medicine, Pediatric Critical Care, Mayo Clinic, Rochester MN, United States

**Keywords:** microcirculation, hemodynamic, videomicroscopy, neonate, children, critically ill

## Abstract

Cardiovascular instability and reduced oxygenation are regular perioperative critical events associated with anesthesia requiring intervention in neonates and young infants. This review article addresses the current modalities of assessing this population's adequate end-organ perfusion in the perioperative period. Assuring adequate tissue oxygenation in critically ill infants is based on parameters that measure acceptable macrocirculatory hemodynamic parameters such as vital signs (mean arterial blood pressure, heart rate, urinary output) and chemical parameters (lactic acidosis, mixed venous oxygen saturation, base deficit). Microcirculation assessment represents a promising candidate for assessing and improving hemodynamic management strategies in perioperative and critically ill populations. Evaluation of the functional state of the microcirculation can parallel improvement in tissue perfusion, a term coined as “hemodynamic coherence”. Less information is available to assess microcirculatory disturbances related to higher mortality risk in critically ill adults and pediatric patients with septic shock. Techniques for measuring microcirculation have substantially improved in the past decade and have evolved from methods that are limited in scope, such as velocity-based laser Doppler and near-infrared spectroscopy, to handheld vital microscopy (HVM), also referred to as videomicroscopy. Available technologies to assess microcirculation include sublingual incident dark field (IDF) and sublingual sidestream dark field (SDF) devices. This chapter addresses (1) the physiological basis of microcirculation and its relevance to the neonatal and pediatric populations, (2) the pathophysiology associated with altered microcirculation and endothelium, and (3) the current literature reviewing modalities to detect and quantify the presence of microcirculatory alterations.

## Introduction

Most clinical and blood pressure tools available to determine circulatory hemodynamic changes in the adult, pediatric, and neonatal populations evaluate macrocirculation as a surrogate of oxygen delivery and adequate end-organ perfusion pressure. The Surviving Sepsis Campaign Guidelines for managing septic shock and sepsis-associated organ dysfunction in children (1) recommend heart rate, capillary refill, and urinary output as clinical markers of cardiac output to assess fluid resuscitation. Gas exchange utilizes capnography, oxygen saturation probes, and blood gas analysis. Near-infrared spectroscopy (NIRS) noninvasively measures oxygen saturation in the vasculature, monitoring tissue perfusion and oxygen delivery (2). Addressing tissue perfusion meeting cellular metabolic demands requires the use of clinical biomarkers such as serum lactate (3, 4), mixed venous oxygen saturation (SmvO_2_) (5), and base deficit (6). Elevated lactate levels are recognized as an indirect marker for tissue hypoperfusion (7). Strategies to evaluate optimal organ resuscitation include serum lactate clearance and capillary refill (8). For the critically ill pediatric population, elevated lactate is consistently associated with mortality (9), including sepsis and septic shock (4). Targeting resuscitation to clear lactate decreases organ dysfunction (3). Although the macrocirculation assessment and monitoring of oxygen delivery is an accepted management strategy, a knowledge gap exists in addressing microcirculation as a treatment target in the resuscitation of the hemodynamically compromised critically ill pediatric patient.

## Microcirculation

**Figure 1:**
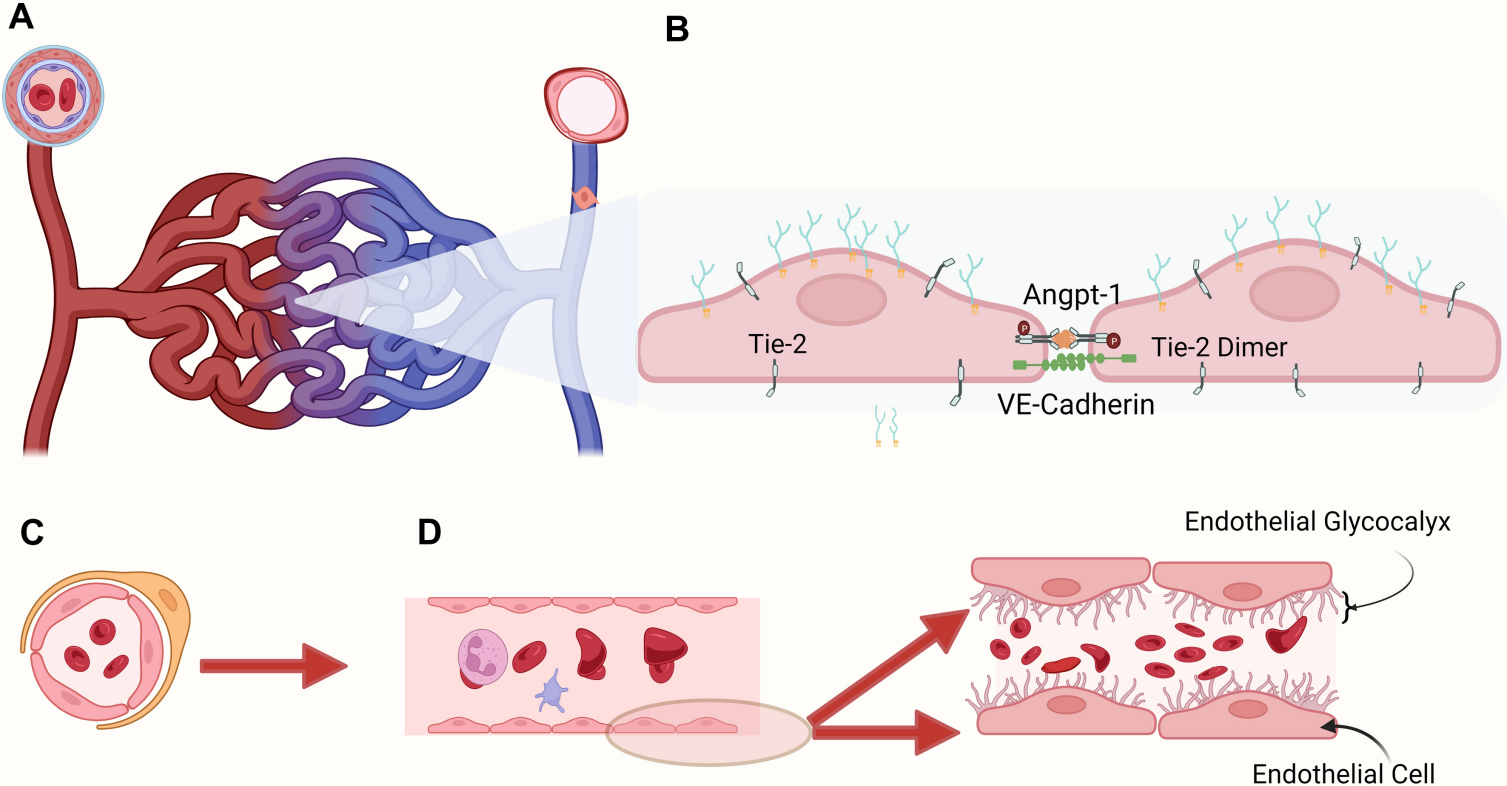
Microvasculature: (**A**) pericytes surround and support the capillaries, the most numerous and dynamic component of the microcirculation where gas and metabolite exchange occurs, and postcapillary venules, where endothelial cells lack tight junctions and are leakier than capillaries. (**B**) Representation of two endothelial cells joined transversally to maintain intact endothelium. (**C**) Cross sectional view of the microcirculation with red blood cells circulating in the inner section (**D**) Graphic representation of the glycocalyx covering the endothelial cells lining the blood vessel. The membrane bound main components include proteoglycans (syndecans, glypicans), glycoproteins (selectins, integrins). The endothelial surface layer includes hyaluronan, plasma proteins, and soluble proteoglycans. Shedding of endothelial glycocalyx (EG) components into the plasma is related to different critically ill conditions, potentially indicating the degree of endotheliopathy.

Adequate tissue perfusion balances oxygen delivery (DO_2_) and oxygen consumption (VO_2_) along with the actual exchange of nutrients and waste products at the microcirculatory level. Anatomically, the microcirculation consists of blood vessels <20 µm in diameter (microvessels), including capillaries, arterioles, and venules (10). The capillaries are dynamic vascular vessels <10 µm between the arterioles containing smooth muscle cells and regulating blood flow and venules (Figure 1). The distribution and magnitude of blood flow is a coordinated interaction between arteriolar, capillary, and venular segments responding to metabolic demands (11). The capillaries within the microcirculation are important distribution centers delivering oxygen and nutrients, signaling molecules, and medication products to tissues and cells. They also support removing waste products and are essential in fluid movement and temperature control (12).

The parallel function of the macrocirculation with the microcirculation has been labeled hemodynamic coherence (13). However, optimal macrocirculation resuscitation does not necessarily reflect adequate microcirculatory perfusion. The dissociation that occurs between the macrocirculatory and microcirculatory circulations has been described in acute pathologic conditions, including shock (14, 15), hypoperfusion (16), cardiopulmonary bypass (17), and sepsis (18, 19) among the most commonly investigated.

Different techniques exist to assess the microcirculation (MC), detailed in Table 1 (10). Capillaroscopy (22) and laser scanning confocal imaging (23, 24) have limited use in the clinical setting. The introduction of handheld vital microscopy (HVM) using an illumination unit with green light (wavelength 548 nm for optimal oxyhemoglobin and deoxyhemoglobin light absorbance) and a light guide with a magnification lens (25) provided a potential venue to noninvasively obtain images useful for clinical assessment and treatment response at the bedside. The analysis and interpretation of HVM data involve two main components of oxygen-carrying capacity: red blood cell flow through the capillaries (oxygen delivery) and the density of the perfused capillaries (diffusive transport of oxygen) (10). Traditional microcirculatory parameters include total vessel density (TVD), functional capillary density (FCD), perfused vessel density (PVD), proportion of perfused vessels (PPV), microcirculatory flow index (MFI), and microcirculatory heterogeneity index (MHI) described in Table 2.

**Table 1:** Types of microcirculation assessment devices.

Technical device	Device	Anatomical site	Characteristics
Handheld Vital Microscopy (HVM) -Orthogonal polarization	OPS	Sublingual	Pressure artifacts can distort the image
-Sidestream dark field	SDF	Buccal mucosa	
-Incident dark field	IDF	Skin Ear	
Laser doppler perfusion imaging (20)	Flowmetry Perfusion		Non-contact measurements Sensitive to motion artifacts
Laser speckle contrast imaging			Non-contact measurements Sensitive to motion artifacts
Capillaroscopy		Nailbed	
SDF software (GlycocCheck©) (21)	Measures Glycocalyx	Sublingual	Measures endothelial glycocalyx thickness Microvascular vessel density Red blood cell filling percentage Reported as perfused boundary region (PBR)

Adapted from (20) and (21)

**Table 2:** Microcirculation variables analyzed after image acquisition.

Variable	Abbreviation	Definition	Description
Proportion of perfused vessels	PPV	Grid-based score (3 horizonal and vertical lines). Percentage of perfused vessel per total number of vessel crossings	Binominal determination of red blood cell velocity: - Flow - No flow
De Backer score	–	Grid-based score (3 horizonal and vertical lines). Vessel crossings per grid length	Proxy of total vessel density, includes different vessel types. Together with PPV proxy of FCD
Microvascular flow index	MFI	Grid-based score per quadrant: 0 = stop flow 1 = intermittent flow 2 = sluggish flow 3 = normal flow	Assesses average red blood cell velocity per quadrant. Good reproducibility.
Total vessel density	TVD	Software supported, Total vessel area per surface area	Presents as an absolute number. Time-consuming analysis requiring manual correction of software supported vessel tracing. Measures vessel diameter.
Perfused vessel density	PVD	Percentage of perfused vessels × TVD	Similar to functional capillary density (FCD). Time consuming analysis
Heterogeneity index	HI	Coefficient of variation. Highest-lowest value/mean	Determines the heterogeneity of blood flow. Provides additional information. Calculation can use MFI or PPV
Functional capillary density (26)	FCD	Sum of the length of all capillaries containing moving RBCs	

Adapted from (10).

Three generations of HVMs have been developed (27). The first generation of HVM involved orthogonal polarization spectral (OPS) imaging (28) using light linearly polarized in one plane and collecting imaging through a second polarizer oriented in an orthogonal plane. A novel technique in HVM was developed in 2007 using sidestream dark field (SDF) imaging, improving quality imaging (29), currently used as a research tool assessing microcirculatory function in the clinical setting and animal models (16, 30). A newer HVM based on incident dark field illumination (IDF), considered a third-generation device, has been introduced in the clinical setting (31). This model combines high-density pixel-based imaging and short-pulsed illumination, providing high-resolution optics (27). Introducing an automatic algorithm software (MicroTools) eased data analysis collected by the HVM (32).

The inner section of the vascular system, the tunica intima, includes the endothelium, where endothelial cells (ECs) line the internal vascular system forming tight junctions, covered by the endothelial glycocalyx (EG), the luminal layer within the blood vessel and fundamental determinant of mechanotransduction and vascular permeability. The vascular endothelium is a highly specialized and physiologically important organ system regulating vascular permeability, vascular tone, cell adhesion, controls blood fluidity, allows macromolecular transfer between blood and tissue, and modulates immune cell recruitment and activation and platelet function (12). While ECs are first-line regulators of proinflammatory and immune responses, their role in neovascularization is also vital in tissue repair (33). The endothelial surface is lined by the endothelial glycocalyx (EG). It is composed of a glycan-rich layer consisting of highly sulfated, negatively charged glycosaminoglycans, including heparan sulfate and chondroitin sulfate attached to the endothelial surface-anchored proteoglycans: syndecans and glypicans (34). Damage to the endothelial glycocalyx can be assessed by screening for serum biomarkers. Recently, in addition to the assessment of the microcirculation, a non-invasive method to measure the size of the endothelial glycocalyx within the vascular microvessels became available, consisting of a handheld non-invasive camera collecting *in vivo* images of blood flow in the capillaries, coupled with the GlycoCheck™ software (35) measuring the thickness of the endothelial glycocalyx.

This review addresses (1) the physiological basis of microcirculation and its relevance to the neonatal and pediatric populations, (2) the pathophysiology associated with endothelial and endothelial glycocalyx dysfunction impacting the microcirculation function, and (3) the current literature reviewing modalities to detect and quantify the presence of microcirculatory alterations using HVM alone or coupled with endothelial glycocalyx assessment.

## Material and methods

Search Strategy: In this review, the focus centers on the technology currently available to monitor microcirculation in the critically ill neonate and pediatric populations and its effectiveness during resuscitation. The template of Preferred Reporting Items for Systematic Reviews and Meta-Analysis (PRISM) (36) was used to collect and categorize the information. A systematic search was conducted in the following databases: PubMed®, MEDLINE OvidSP, and Google Scholar. Single and paired combinations of terms included “microcirculation”, “videomicroscopy”, and “children”. This review focuses on data published between 2010 and 2023. The data extraction was conducted by one investigator (GMA). The initial screening was completed independently from each other, and duplicates were deleted. The studies were merged for a second analysis. Eligibility and inclusion criteria encompassed prospective observational studies, cohort studies, systematic reviews, comprehensive reviews, and clinical trials. Screening criteria for inclusion included the following parameters: (1) Age group: the preterm, neonate, and children up to the age of 18 years, (2) the period reviewed covers the years 2010 and 2023 inclusively, (3) clinical condition labeled as critically ill or hemodynamically unstable, and (4) assessment of the microcirculation with handheld vital microscopy (HVM) ± assessment of the endothelial glycocalyx. We selected most of the reports with control cohorts in the prospective observation groups. We did not include studies describing the endothelium or endothelial glycocalyx assessment alone, only those combined with HVM assessments of the microcirculation. The results were qualitatively analyzed. The heterogeneity of patient populations, the small number of subjects in the reports, and the variety of techniques used for assessment challenged a quantitative analysis.

## Results

**Figure 2:**
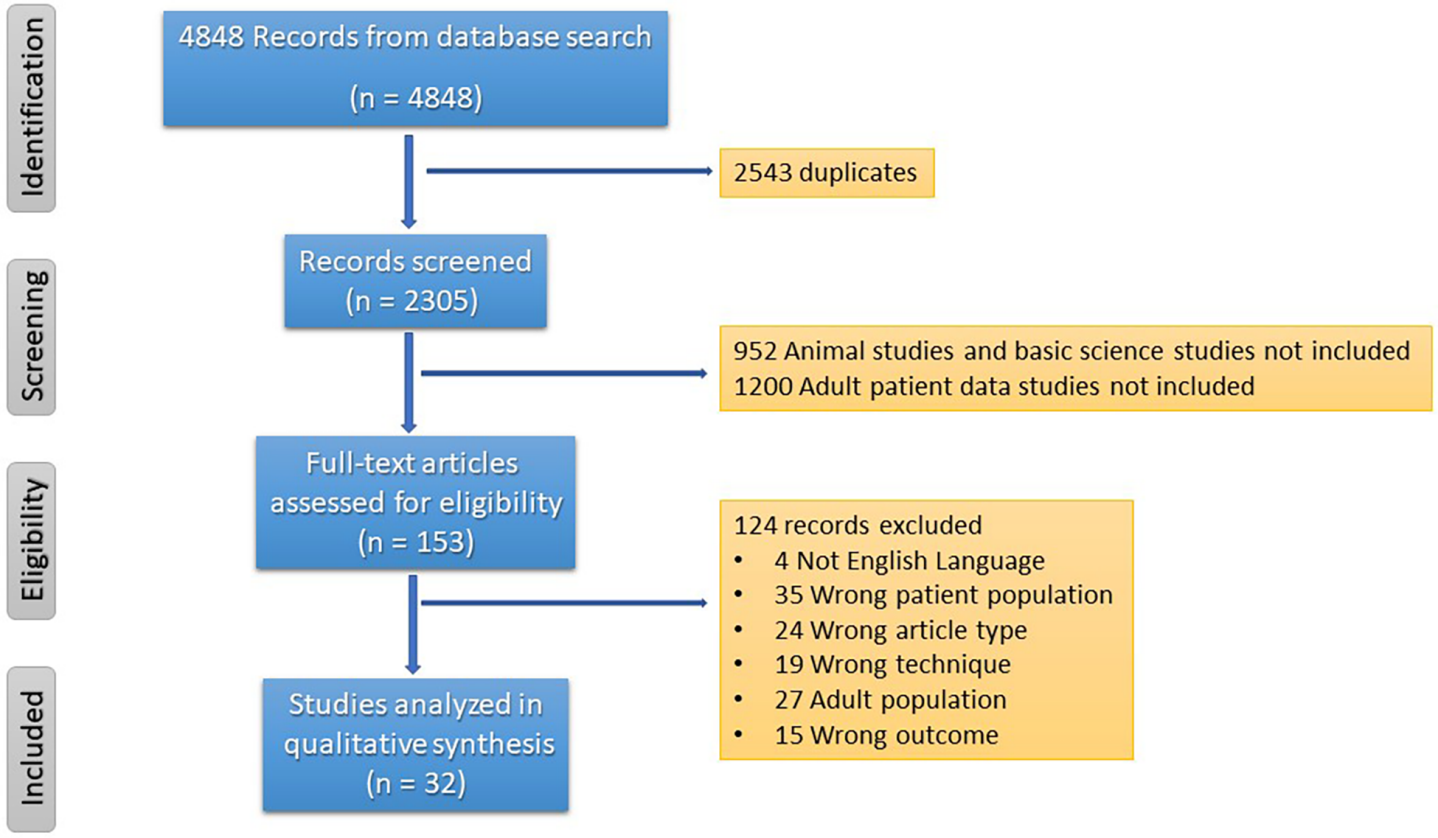
Flow diagram and selection of eligible manuscripts included in this report.

The number of pediatric and neonatal studies evaluating microcirculation using HVM in healthy and diseased children is minuscule compared to the available information published for the critically ill adult population. Entering the terms described in the methods section, we obtained 1,494 manuscripts from PubMed.gov, 1,430 references from Google Scholar, and 1,924 more from OVID Medline. The next step included the deletion of duplicate data (2,543 references). Abstracts from 2,305 studies were screened, and 153 full-text articles met the requirements for a full-text review. From this group, 32 manuscripts described the use of video microscopy in critically ill pediatric and neonate patients. This review describes the critically ill pediatric population of 16 pediatric (Table 3) and 11 neonatal (Table 4) reports. The working flowchart is presented in Figure 2. Most studies describe prospective observational reports involving a small number of patients. We also include five reviews evaluating microcirculation with and without endothelial biomarkers (Table 5). Maitoza et al. published the only systematic review using HVM in critically ill neonates and children and described 27 studies (64). In addition, two reviews of the literature describe the non-invasive measurements of the MC using HVM devices (OPS, SDF, and IDF) in neonates and pediatric populations (65) and the use of OPS and SDF in neonates and pediatrics (66). Top et al. reviewed nine studies using OPS and SDF in neonates and pediatric patients (67). Lastly, Puchwein-Schwepcke et al. published a mixed review of HVM combined with serum endothelial glycocalyx biomarkers (68).

**Table 3:** Summary of critically ill pediatric studies using handheld vital microscopy (HVM).

Reference	Critical illness	Patient (*n*)	Groups (*n*)	HVM	Site	Intervention	Outcome
Top et al. (37)	Septic Shock	18	Survivor: 15 Non-survivor: 3	OPS	Buccal mucosa	Fluids Vasopressor Inotropes	Survivors: • FCD increased 48 h • MFI improved Non-survivors: • FCD no change • MFI no change
Paize et al. (38)	MCD	60	MCD: 20 Control: 40 -Anesthesia 20 -Awake 20	SDF	Subling	Mechanical ventilation and management for MCD, anesthesia for routine procedures	The study combined HVM with endothelial biomarkers. Decreased MFI, PPV, and PPD in MCD. Initial MFI predicted the duration of mechanical ventilation in MCD. Altered MC correlated with endothelial biomarkers levels.
Scolletta et al. (39)	CHD	24	Cyanosis: 7 Non-cyanotic 14	SDF	Subling	Anesthesia, CPB, RBC management	TVD, PPV, PVD, and MFI are different between cyanotic and non-cyanotic patients, Increased PPV observed over time in the cyanotic group.
Gonzalez et al. (40)	CHD	30	Cyanosis: 14 Non-cyanosis 16	SDF	Subling	Anesthesia in CHD repair	Cyanotic: • High TVD Cyanosis increases vascular density. Younger patients have lower MFI.
Gonzalez et al. (41)	PICU	18	T1 = 15 T2 = 9 T1 and T2 = 6	SDF	Subling	PICU admission for more than 24 h	Patients evaluated on admission (T1) and day 3 (T3). No correlation with PICU length of stay, mechanical ventilation, vasoactive drug therapy or ECMO. Small number of patients.
Gonzalez Cortes et al. (42)	CHD	24	All congenital heart disease	SDF	Subling	CPB	Microcirculatory parameters worsened during CPB and returned to baseline after surgery. Children with CHD have higher small vessel density and higher density of perfused small vessel at baseline; lower MFI and higher heterogeneity during surgery.
Erdem et al. (43)	ECMO	34	VV ECMO 12 VA ECMO 22	IDF	Subling	Data collected before and during ECMO	No effect was observed in the ECMO populations using HVM before or during treatment
Erdem et al. (44)	CHD	73	CHD, CPB 38 Elective, non-cardiac 35	IDF	Subling	CHD and surgery with CPB compared to non-cardiac surgical patients	Patients with CHD have decreased microcirculatory perfusion and higher small densities compared to control group. After CPB, microcirculation is further impaired.
Nussbaum et al. (45)	CHD	55	CPB CHD: 36 -CHD, no CPB 4, Control: 15 -Cath: 6, -Cleft palate: 9	SDF + Glyco check soft-ware	Ear conch	CPB	Decreased MFI and PVD in CPB after heart surgery. CPB induces endothelial glycocalyx thickness and microvascular perfusion dysregulation.
Buijis et al. (46)	Cardiac Arrest (CA)	40	CA, ROSC: 20 Control: 20	SDF	Buccal mucosa	Therapeutic Hypothermia (in CA)	PVD and MFI lower in non-survivors starting hypothermia. HVM for possible prognostication in CA ROSC.
Schinagl et al. (47)	Anemia	37	Anemia: 19 Control: 18	SDF	Buccal mucosa	Red blood cell transfusion	TVD was lower and RBC velocity higher pre-transfusion. Microcirculation improved after transfusion.
Fernandez et al. (48)	MIS-C	3	MIS-C: 2 Control: 1	SDF + Glyco check soft-ware	Subling	Vasoactive support and steroid treatment	PBR (perfused boundary region) demonstrated endothelial glycocalyx damage in patients with MIS-C.
Fernandez et al. (49)	Sepsis Septic shock	106	Balanced fluid resuscitation: 48 Unbalanced fluid resuscitation: 58	SDF + Glyco check soft-ware	Subling	IV fluid resuscitation	Children with sepsis have worsening endothelial glycocalyx dysfunction when unbalanced crystalloid boluses are used for resuscitation (normal saline) compared to balanced fluids such as lactate ringers.
Hilty et al. (50)	Periop and critical illness	267	Control: 40 Pediatric P: 10 Adult P: 72 Adult ICU: 145	SDF + AVA software	Subling	MicroTools software assessment	MicroTools software validation over a wide range of perioperative and critically ill patient populations using data-mining
Lyimo et al. (51)	Malaria	119	Control: 31 Severe malaria: 69 Other: 19	IDF + software to calculate PBR	Buccal mucosa	Standard malaria treatment	PBR was significantly increased in patients with severe malaria (thin glycocalyx). Plasma elevation of heparan sulfate, syndecan-1, HA, GAG (glycocalyx). Elevation angiopoietin-2, thrombomodulin, endothelin-1 (endothelium).
Wagner et al. (52)	Major surgery thorax & abdomen	11	No control Major surgery: 11	SDF + AVA + Glyco check	Subling	Perioperative monitoring in high-risk surgery Fluid resuscitation, Norepinephrine	Capillary density reduced after surgery started. Microvascular flow and serum glycocalyx markers (syndecan-1 and hyaluronan) increased. PBR

FCD, functional capillary density; MFI, microvascular flow index; CHD, congenital heart disease; SR, systematic review; OPS, orthogonal polarized spectra; SDF, sidestream dark field; IDF, incident dark field; CAVM, computer-assisted video microscopy; DRS, diffuse reflectance spectroscopy; CBP, cardiopulmonary bypass; PICU, pediatric intensive care unit; RBC, red blood cell; MCD, meningococcal disease; MC, microcirculation; PRISM, pediatric risk of mortality; LTH, local thermal hyperemia; ROSC, return of spontaneous circulation; CDH, congenital diaphragmatic hernia; MIS-C, multisystem inflammatory syndrome-COVID; IDF, incident dark-field imaging; HA, hyaluronic acid; GAG, sulfated glycosaminoglycan.

**Table 4:** Summary of critically ill neonatal and premature population studies using handheld vital microscopy (HVM).

Reference	Critical illness	Total patient (*n*)	Groups (*n*)	HVM	Site	Intervention	Outcome
Alba et al. (53)	Suspected infection	47	Newborn: 47 -Infection: 16 -No infection 31	OPS	Ear conch Skin/arm	Infection treated with antibiotic	The proportion of vessels with continuous flow is lower in infants with infection
Ergenekon et al. (54)	Polycytemia	15	CA, ROSC: 20 Control: 20	SDF & NIRS	Skin/arm & head and calf	Partial exchange transfusion	TVD showed no difference after treatment, unlike MFI of small and total vessels, which were higher. Cerebral tissue oxygenation (cTOI) was significantly higher.
Top et al. (55)	Persistent Pulmonary Hypertension (PPHN)	8	Neonates 6 Pediatric 2	OPS	Buccal mucosa	Nitric Oxide use for CDH, Meconium aspiration, bronchiolitis, PARDS	iNO improves the functional capillary density in the microcirculation
Ergenekon et al. (56)	Hypoxic ischemic encephalopathy (HIE)	14	HIE: 7 Control: 7	SDF	Skin/axilla	Therapeutic Hypothermia (TH)	There is a significant decrease in microcirculatory blood flow in patients with hypothermia.
Hiedl et al. (57)	Patent Ductus Arteriosus (PDA)	25	Preterm <35 weeks GA -PDA 13 -control 12	SDF	Skin/axilla	Indomethacin/Ibuprofen	Fewer large vessels and significantly more small vessels in the PDA group. After treatment, these differences disappear.
Top et al. (58)	Respiratory failure	28	Respiratory failure -ECMO 21 -Ventilated 7	OPS	Buccal mucosa	ECMO	ECMO prevents further deterioration of the microcirculation in patients with respiratory failure started on ECMO
Buijs et al. (59)	CDH	56	CDH: -CDH 28 -Control 28	SDF	Buccal mucosa	Catecholamine support	Better microcirculation in the control group. The use of catecholamines does not improve microcirculation in CDH
Schwepeke et al. (60)	Hypotension premature <30 weeks BW < 1,225 g	21	Hypotensive 10 Control 11	SDF	Right arm	Catecholamine support	Patients followed prospectively. FVD was higher in the hypotensive group after birth and recovered 12 h later
Fredly et al. (61)	Neonatal asphyxia	28	Prospective evaluation	LDPM CAVM DRS	Skin (chest)	Therapeutic Hypothermia (TH)	Day 1 and 3 during hypothermia and day 4 after rewarming. Capillary flow velocity was reduced, and tissue oxygen extraction was higher during TH.
Fredly et al. (62)	Neonatal asphyxia and elevated CRP	28	Low CRP 18 High CRP 10	LDPM CAVM DRS	Skin (chest)	Rewarming after TH	High CRP is associated with higher LDPM perfusion, lower functional vessel density, and larger heterogeneity of capillary flow velocities.
Puchwein-Schwepcke et al. (63)	Prematurity and permissive hypercapnia	12	High pCO2: 5 Control: 6	SDF	Skin (right arm)	Extremely low-birth-weight (ELBW) (<1,000 g) Mechanical ventilation wean	Permissive hypercapnia affects the microcirculation characterized by decreased FVD

FCD, functional capillary density; MFI, microvascular flow index; CHD, congenital heart disease; SR, systematic review; OPS, orthogonal polarized spectra; SDF, sidestream dark field; IDF, incident dark field; LDF, laser doppler flowmetry; LDPM, laser doppler perfusion measurement; CAVM, computer-assisted video microscopy; DRS, diffuse reflectance spectroscopy; RF, respiratory Failure; CBP, cardiopulmonary bypass; RBC, red blood cell; MCD, meningococcal disease; MC, microcirculation; LTH, local thermal hyperemia; ROSC, return of spontaneous circulation; CDH, congenital diaphragmatic hernia; CRP, C-reactive protein; PBR, perfused boundary region.

**Table 5:** Pediatric reviews using handheld vital microscopy (HVM) to assess microcirculation.

Reference	Critical illness	Studies included (*n*)	Groups (*n*)	HVM type (*n*)	Site	Intervention	Outcome
Maitoza et al. (64)	SR critical illness	27	Studies included: Pediatric 7 Neonate 20	OPS 6 SDF 11 IDF LDF 6 NIRS 1 LDPM, CAVM, DRS 2	Oral mucosa Forehead Ear conch Cutaneous Buccal mucosa Sublingual	Critical care support	Critical illness impacts hemodynamic coherence. Microvascular variables do not parallel hemodynamic parameters.
Erdem et al. (65)	Review	34 S	Neonate 22 Pediatric 12	OPS 11 SDF 19 IDF 4	Cutaneous	Critical care support	HVM is a non-invasive tool capable of monitoring microcirculation. There is a lack of evidence for therapeutic clinical decision-making and its impact on clinical outcomes.
Top et al. (66)	Review	9 S	Neonate 6 Pediatric 3	OPS SDF	Skin Buccal mucosa	Critical care support	Lack of restoration of deranged MC has a better predictive value for mortality than PRISM.
Kuiper et al. (67)	Review	23 S	Neonate 16 Pediatric 7	OPS SDF	Skin Sublingual Ear	Critical care support	Describes the use of HVM in pediatric critical care settings, perspectives and research opportunities.
Puchwein et al. (68)	Mixed review HVM and biomarkers	14 S	CHD 6 Trauma (peds) 2 ID and Immune 5 Diabetes 1	HVM GlycoCheck Biomarkers	Serum for biomarker Multiple and PBR (IDF imaging)	Critical care support	Describes mostly biomarkers used to detect glycocalyx damage with two studies mentioning HVM

FCD, functional capillary density; MFI, microvascular flow index; CHD, congenital heart disease; SR, systematic review; OPS, orthogonal polarized spectra; SDF, sidestream dark field; IDF, incident dark field; LDF, laser doppler flowmetry; LDPM, laser doppler perfusion measurement; CAVM, computer-assisted video microscopy; DRS, diffuse reflectance spectroscopy; CBP, cardiopulmonary bypass; RBC, red blood cell; MCD, meningococcal disease; MC, microcirculation; PRISM, pediatric risk of mortality; LTH, local thermal hyperemia; ROSC, return of spontaneous circulation; CDH, congenital diaphragmatic hernia; MIS-C, multisystem inflammatory syndrome-COVID.

A summary in Table 1 describes the multimodal techniques used for HVM. The methodology used by the investigators varied depending on the age group investigated and the HVM device used. The premature and neonatal populations showed the most anatomically diverse locations for microcirculation screens. Frequently reported regions for assessment reported in the premature and neonatal populations included the upper arm, nailbed, and ear conch. The buccal mucosa and the sublingual approaches were challenging to screen in the non-cooperative neonate and pediatric subjects, resulting in discomfort for the patient and poor image quality collection. Table 2 describes the measurement variables commonly reported using handheld devices adapted.

A summary of the details of the studies, findings, disease process, and conclusion is shown in a table format. Table 3 describes the pediatric group, and Table 4 describes the neonatal population findings. Table 5 includes a collection of recent reviews using HVM in the pediatric population, and one of the reports describes the use of HVM to examine microcirculation coupled with endothelial glycocalyx assessment.

## Sepsis

### Assessment

Top et al. (37) used OPS to assess microcirculation characteristics 24 h after admission to the pediatric intensive care unit (PICU) for patients not surviving sepsis. The patients diagnosed with septic shock required fluid resuscitation, vasopressor/inotropes, and mechanical ventilation. On the first day, there were no differences in functional capillary density (FCD) between groups. Twenty-four hours later, the FCD significantly increased, and the microvascular flow index (MFI) improved in the survival group, suggesting that persistent microcirculatory abnormalities potentially lead to multiorgan system failure and death. Adult patients with sepsis demonstrate almost similar findings (69). A neonatal population study addressing infection showed a reduction in the proportion of vessels with continuous flow using the ear conch location with OPS (53). In meningococcal disease (MCD), Paize et al. compared 20 affected patients with 20 patients undergoing anesthesia for surgical procedures and 20 awake patients (38). This group used SDF in the sublingual location and measured microcirculation variables: microvascular flow index (MFI), capillary density (CD), proportion of perfused vessels (PPV), and perfused vessel density (PVD) in parallel to the endothelial biomarkers: intracellular adhesion molecule-1 (ICAM-1), vascular cell adhesion molecule-1 (VCAM-1), E-selectin and P selectin. The patients with MCD were followed through their Pediatric Intensive Care Unit (PICU) stay. The authors found microcirculatory abnormalities correlating with biomarkers of endothelial dysfunction, and these abnormalities improved with clinical recovery. On a separate patient population, Fernandez-Sarmiento et al. published two cases with SARS-CoV-2-related multisystem inflammatory syndrome (MIS-C) investigating the endothelial glycocalyx using HVM with the Glycocheck™ software and plasma EG biomarkers revealing elevated plasma and imaging biomarkers of endothelial activation and endothelial glycocalyx degradation (48). Patients affected by severe malaria caused by Plasmodium falciparum were studied by Lyimo et al. (51). Using IDF of the buccal mucosa in patients with severe malaria compared to a control group, patients suffering from severe malaria demonstrated alterations in microcirculation and the endothelial glycocalyx.

### Interventions

Published work assessing medical interventions in shock management using HVM in combination with endothelial biomarkers and glycocalyx assessment has revealed that fluid administration and catecholamine use can be evaluated using HVM (26). A recent pediatric report demonstrated that fluid choice during resuscitation could have an unwanted impact on the endothelial system (49). This group compared a balanced solution for resuscitation with an unbalanced solution. The group used SDF with the GlycoCheck™ software and the endothelial serum biomarkers: angiopoietin-2 levels for vascular permeability and annexin A5 for apoptosis to determine microcirculation and endothelial responses. Unbalanced solutions increased endothelial degradation 6 h after fluid resuscitation compared to balanced solutions. These findings were also associated with a more significant elevation of biomarker levels and greater odds of metabolic acidosis and acute kidney injury returning to baseline levels 24 h later.

## Congenital heart disease

Several reports describe parameters obtained using HVM in neonates and children with congenital heart disease. Nussbaum and colleagues investigated the impact of cardiopulmonary bypass (CPB) on the microvasculature and endothelial glycocalyx using SDF and the GlycoCheck™ software. The microcirculation data demonstrated an acute reduction of the microvascular perfusion after cardiac surgery with CPB, particularly with aortic clamp and deep hypothermic cardiac arrest (showing decreased MFI and PVD). The perfused boundary region (PBR) increases as the glycocalyx is damaged. Immediately after surgery and CPB, PBR was increased, suggesting glycocalyx degradation. Nussbaum et al. found a relationship between changes in PBR and MFI and the need for mechanical ventilation and catecholamine support with longer CPB times (45). These results align with the study published by Bruegger et al. (70), where endothelial biomarkers Syndecan-1 and hyaluronan increased during, and after cardiac surgery. Similarly, in a more recent report, Erdem et al. (44) described the microcirculation in children with congenital heart disease before and during their cardiac surgical procedure and compared the microcirculation with a group of pediatric patients without heart disease undergoing surgery. Their findings showed decreased microcirculation perfusion and high vessel density in patients with congenital heart disease, with increased capillary recruitment. Specifically, both groups have similar perfused vessel densities and red blood cell velocities. However, children with CHD have less perfused vessels, lower perfusion quality, and higher small vessel densities.

More data addressing CHD has supported these findings. Scolletta et al. studied microcirculation in children with cyanotic and non-cyanotic heart disease. This group used HVM SDF sublingually in 24 children, 7 of them with cyanotic heart disease, and found that microcirculatory variables (PPV, TVD, and PVD) were different between patients with cyanosis and those without cyanosis (39). Gonzalez et al. included 14 patients with cyanosis and 16 without cyanosis and assessed the microcirculation after anesthesia induction using sublingual SDF (40). Their data demonstrated a higher TVD with an increase in small blood vessels in the sublingual microcirculation, possibly related to the chronic hypoxia in patients with cyanosis, potentially a mechanism of adaptation. Gonzalez Cortes et al, using SDF-HVM, found that children with CHD have higher small vessel density and higher density of perfused small vessels at baseline; and lower MFI and higher heterogeneity during surgery in 24 patients undergoing surgery under cardiopulmonary bypass surgery (42).

The data obtained from these different manuscripts show that patients with CHD have differences in microcirculation compared to patients with normal cardiac anatomy and physiology.

## Perioperative

The concept of using HVM as a tool in the perioperative period was described by Hitly et al. These investigators used HVM data from 267 adult and pediatric patients undergoing surgery, diagnosed with sepsis and heart failure, and included healthy volunteers to validate a software algorithm to ease the HVM analysis (MicroTools) (50). The algorithm-based analysis of the sublingual microcirculation closely matched the manual analysis collected from the patient variety. The pediatric population included data from 120 perioperative patients undergoing elective cardiac surgery. The data was collected after anesthesia induction and included total vessel density (TVD) and functional capillary density (FCD). The authors did not specifically address the pediatric-specific data. However, in general, the authors found a strong correlation between the manual- vs. algorithm-based measurements in FCD, potentially suggesting that in the pediatric population, the algorithm-based measurements are reliable. Monitoring pediatric patients during high-risk surgeries depends on optimizing the macrocirculation. In a study completed by Wagner et al. (52), the authors addressed the feasibility of monitoring the microcirculation using handheld vital microscopy in a small group of pediatric patients. The microcirculation was assessed at four-time points during the perioperative period and was coupled with serum glycocalyx markers (syndecan-1 and hyaluronan). This effort was challenged by the number of personnel and logistics associated with its routine use in this clinical setting. In this small population, the findings showed microvascular changes and direct injury to the glycocalyx.

## Anemia

Schinagl et al. (47) explored the effects of blood transfusion on microcirculation. In a group of 19 patients with anemia, the authors found a lower TVD and higher RBC velocity than control patients. After blood transfusions, TVD increased with a concomitant decrease in RBC velocity in medium-sized vessels. Although these changes occurred in the patients with anemia after receiving RBCs, TVD and RBC velocities did not reach the control group values. From the 19 patients in this study, a subgroup analysis described nine patients with anemia and sepsis. These patients showed a lower TVD before transfusion, with a larger increase after transfusion than patients with anemia and without infection. The data was collected with HVM SDF assessing the buccal microcirculation. The control group included matched patients of age and sex receiving minor reconstructive surgery and without medical problems.

## Cardiac arrest

Buijs et al. studied the microcirculation after the return of spontaneous circulation (ROSC) in patients who suffered cardiac arrest (46). They used HVM SDF to investigate the microcirculation at the buccal mucosa during and after therapeutic hypothermia (TH). The prospective study covered four years, including 20 pediatric patients with cardiac arrest and age- and gender-matched control normothermic children without cardiorespiratory pathology. The goal for temperature in TH was set at 34.0°. During TH, all variables in the post-cardiac arrest group were lower than the control group and did not differ after returning to normothermia. Microcirculatory impairment was significantly present in the non-survivor group at the beginning of TH, and it is proposed that using non-invasive microcirculatory monitoring can be useful to the clinician for prognostication purposes. In a separate study including neonates, Ergenekon et al. used HVM SDF imaging from the axillary area for a group of 7 newborns who suffered hypoxic-ischemic encephalopathy (HIE) and underwent TH and head cooling (56). Microcirculatory blood flow significantly decreased during hypothermia, becoming similar to the control group after rewarming. Fredly et al. (61) used laser Doppler perfusion measurements (reduced during hypothermia) and diffuse reflectance spectroscopy (DRS) to assess microvascular oxygen extraction. The authors evaluated the capacity for oxygen delivery. They described a mean functional capillary density (FCD) higher during cooling and after rewarming in the group with HIE, along with a significant increase in oxygen extraction. Skin microcirculatory responses significantly differed after rewarming in a subgroup of patients with HIE and elevated CRP (62).

## Respiratory failure

Top et al. investigated the effect of inhaled nitric oxide on neonates with respiratory failure using OPS in oral mucosa at two-time points: 1 h before and 1 h after the initiation of inhaled nitric oxide. Inhaled nitric oxide improved systemic microcirculation in patients with hypoxemic respiratory failure in the newborn population affected with pulmonary hypertension (55). Neonatal microcirculation was examined using HVM OPS imaging in patients with respiratory failure before extracorporeal membrane oxygenation (ECMO) and after initiating ECMO and compared with patients who remained on mechanical ventilation not supported with ECMO (58). These investigators noticed there was no change in microcirculation parameters with ECMO initiation. However, ECMO improved the microcirculation parameters compared to patients with respiratory failure and mechanical ventilation not receiving ECMO. In a more detailed investigation in patients treated with veno-venous (VV) and veno-arterial (VA) ECMO, Erdem et al. (43) aimed to determine the effects of ECMO in the sublingual microcirculation in pediatric and neonatal populations. Their data collection included the Pediatric Logistic Organ Dysfunction 2 (PELOD-2) score, the inotrope score (IS), and the vasoactive-inotrope score (VIS) for clinical data collection. They found no difference. They found that the microcirculatory parameters were not significantly different between VV and VA ECMO, and these parameters were no different in patients with CHD. Also, these parameters were not different between survivors and non-survivors. The authors discuss several possibilities for these findings, including starting ECMO in patients in extremis where the compensatory mechanisms for adequate microcirculation might not have been exhausted. Potentially, a possible explanation might include the heterogeneity of patients included in the study and not rendering adequate sample sizes for the diversity of pathologies included. The comparison in findings between these two manuscripts is challenged by the difference in patient populations included. The neonatal population tends to have more uniform pathologies than the pediatric population. Further studies with more homogeneous populations are needed to determine the effects of ECMO in the microcirculation.

## Premature and neonatal populations

HVM has been used to examine the microcirculation of premature and neonatal disease processes. Polycythemia requiring partial exchange transfusion showed no difference in TVD and higher MFI in total vessels with increased cerebral tissue oxygenation (54). The use of indomethacin in preterms with patent ductus arteriosus (PDA) was reported by Hiedl et al. (57) demonstrating fewer large vessels in the microcirculation of infants with PDA, with increment after medical treatment. Buijs et al. examined 28 newborns diagnosed with congenital diaphragmatic hernia (CDH). A subgroup was receiving vasopressor support and was compared to healthy newborns (59). Catecholamine support in patients with CDH improved the macrocirculatory parameters without improving the microcirculation. The severity of microcirculation dysfunction also predicted a poor clinical outcome and the need for extracorporeal membrane oxygenation (ECMO). VLBW patients with hypotension have higher functional vessel density when compared to a control group, a finding that resolves 12 h after birth (60). Puchwein-Schwepcke et al. studied the effects of hypercapnia in the extremely low birth weight (ELBW) premature population with weight <1,000 gms (63). The investigators noticed a significantly and progressively decreased functional vessel density (FVD), suggesting impaired peripheral microcirculation at the skin capillaries.

## Study reviews in pediatric patients

Several comprehensive reviews have summarized our current knowledge of the use of HVM in critically ill pediatric patients, detailed in Table 5. A recent report combined this approach with the assessment of the endothelial glycocalyx. The systematic review from Maitoza et al. includes microcirculation assessment information using HVM with literature published before 2020 (64). The authors observed several limitations in the manuscripts published, including study design, high subject dropout rate, and a need for standardized normal values for the investigated age populations. The authors highlight the need for future studies to define normal pediatric flow variables and more information describing treatment impact on the pediatric and neonatal microcirculation. Similar conclusions were echoed in the review completed by Erdem et al. (65). These authors highlight the advances in pediatric and neonatal microcirculation assessments, including the description of microcirculation differences between age groups, the presence of abnormal microcirculation in various disease processes despite maintaining normal macrocirculation values, and the persistent microcirculation abnormalities in patients with higher risk for mortality, emphasizing the need for age-related reference values. Kuiper et al. published a review highlighting the role of microcirculation monitoring in patients who later became hemodynamically unstable (66). Of interest to the pediatric critical care setting, Top et al. in 2011 (67) described a collection of reviews in neonates and pediatric patients, proposing microcirculation assessment as an essential hemodynamic variable to include during the evaluation and daily examination of the critically ill pediatric patient. In this review, the group emphasized the change in functional capillary density (δFCD) within the first two days in children affected by septic shock and compared those findings with the pediatric risk of mortality (PRISM). Their results described a better sensitivity and specificity for δFCD, previously reported by these authors as ΔFCD (37). More recently, Puchwein-Schwepcke et al. published a review where the endothelial glycocalyx (EG) was examined using HVM in combination with endothelial biomarkers (68). Their study describes the advances in microscopic technology to assess *in vivo* EG using a recently developed automated acquisition and analysis approach in combination with the determination of EG components as biomarkers: syndecan-1, chondroitin sulfate, hyaluronan, and heparan sulfate from serum and urine levels. In their review, the authors describe the tools to measure the EG and discuss the perfused boundary region (PBR), a variable used to measure the luminal part of the EG accessible to flowing erythrocytes, a concept validated in the adult population (35). This review summarizes the studies related to the physiological development of the EG, its assessment in pediatric clinical studies, and the challenges encountered in the pediatric population, particularly preterm newborns, including the physiology of the blood vessel development in the fetus and neonates. Disease processes reported in this review included cardiopulmonary bypass effects, trauma, infectious diseases, and chronic disorders such as diabetes mellitus. The authors conclude that damage to the EG is well documented in acute and chronic conditions within the adult population. The data for the pediatric and neonatal groups is scant at present. More investigations are needed to characterize the normal development of the EG from the fetus to adulthood, its contribution to a variety of disease processes, and the potential of developing target-specific therapies that can potentially preserve and heal the EG impacting clinical outcomes.

## Discussion

The current information related to HVM highlights the advances and challenges encountered in applying this technology to the neonatal and pediatric population. The evidence varies in the quality of information published and the number of patients used in each report, some of which do not have a control group. Using different techniques and devices resulted in obtaining measurement variables that require consistency among the reports. Videomicroscopy requires understanding the basic technical and photometric information for optimal image acquisition. Massey et al. described the parameters needed for high-quality data collection and the challenges with using them in the adult group (27). Unlike the pediatric population, a more standardized approach exists for the adult population. A task force involving international expert opinion published guideline recommendations for video microscopy targeting the critically ill adult population (10). The consensus discussed handheld videomicroscopy's technology, physiology, variable measurements, and clinical utility. This report has allowed reliable data collection for healthy individuals and patients with different disease processes. The resulting statements regarding the acquisition and interpretation of the microcirculatory images included databases of measurement variables recommended for the different types of shock and the type of evaluation needed for a variety of therapeutic interventions, mainly fluid administration, vasopressor administration, and weaning from ECMO or IABP. The extrapolation of these recommendations to the pediatric group does not apply to the nature of the device and the site needed to collect the data. The authors mention the need to describe normal values for different age groups in pediatrics and the variety of developmental changes occurring in the neonatal population, particularly during the first week of life, requiring further characterization.

The sublingual approach is the ideal location in the adult population to measure microcirculation (10). In a control group, Paize et al. (38) used awake patients older than six years of age for their control group and anesthetized patients younger than six years of age, demonstrating the difficulty of using sublingual SDF in the younger not-sedated population. The degree of abnormality in the microcirculatory flow pattern relates to the severity of the illness and improves over time as the clinical condition resolves. The standardized use of the sublingual approach to measuring microcirculation in the pediatric population and the validity of the norm values for neonates and children are areas of intense research (10, 50). Children require anesthesia or sedation to obtain meaningful measurements, and these values have greater reliability when pediatric patients are anesthetized, endotracheally intubated, and with mechanical ventilation. Cooperation from adult patients using this location is feasible and easily obtained.

The progression in our understanding of microcirculation and the endothelial system has led to the development of new approaches investigating the health of the endothelial glycocalyx (EG). The EG comprises proteoglycans and glycosaminoglycans and is crucial in maintaining a functional barrier and a healthy microcirculation (71). The disruption of this structure is currently recognized as having a central role in several critical diseases, such as sepsis and acute inflammation (72), acute respiratory distress syndrome (73), trauma (74), cardiopulmonary bypass (45), and ischemia/reperfusion (75). Jacobs et al. summarized the microcirculation's physiology and pathophysiology, emphasizing the EG's important role in hemodynamic responses (76). Several recent reviews address the importance of identifying biomarkers that can identify the shedding of the EG in disease processes leading to identifying, prognosticating, and ideally guiding clinical decision-making (68). Understanding the molecular basis of the EG in health and disease sets the possibility of developing new EG targeted therapies. The importance of the endothelium-specific Angiopoietin/Tie2 system controlling endothelial activation and its role in critical illness (77, 78) has received recent attention. The disruption of the endothelial Tie2 system appears associated with coagulopathy triggered by sepsis. Angiopoietin-1 (Angpt-1) binds to Tie2 at the endothelial surface and maintains adhesion between endothelial cells (Figure 1). During inflammation, Tie2 activation decreases, and TIE2 transcription is attenuated. Richter et al. described the association of plasma angiopoietin-1 and angiopoietin-2 levels and the Angiopoietin-2/-1 ratio in critically ill children with sepsis to measure organ injury (79). The authors found that in the acute phase of sepsis, angiopoietin-1 levels are decreased compared to controls, and angiopoietin-2 levels and ratio are elevated. These values correlated with organ injury, impacting mechanical ventilation duration and PICU length of stay. The authors suggest that angiopoietin dysregulation occurs early in sepsis and is potentially related to multiple organ dysfunction. In this review, the authors present data indicating that the Angpt/Tie2 system can be used to diagnose and treat critically ill patients. Few pediatric studies have used angiopoietin serum levels to assessEG degradation. Recently, Fernandez-Sarmient et al. used HVM and syndecan-1 to assess microcirculation and EG integrity along with angiopoietin-2 and annexin A5 levels to determine the effect of balanced and unbalanced crystalloid resuscitation during sepsis (49).

In the adult population, sepsis has been an area where microcirculation derangement has been well described in the literature (80). These data have shown that microcirculatory dysfunction is an early indicator of tissue hypoperfusion and precedes the onset of multiorgan failure and death (81). In 2018, four different types of alterations were described during the 2nd consensus on the assessment of sublingual microcirculation in critically ill patients. These included: Type 1, complete stagnated capillaries (cardiac arrest); Type 2, reduction in the number of flow in capillaries (hemodilution); Type 3, vessels with no flow next to vessels with flowing cells (sepsis, hemorrhage); Type 4, hyperdynamic flow within capillaries (hemodilution, sepsis) (10). For sepsis, the heterogeneity index is significantly elevated. Sepsis and septic shock show significant abnormalities in microperfusion parameters using HVM. In contrast, the data available for the pediatric population to assess microcirculation and endotheliopathy is limited.

Microcirculatory parameters show differences between patients with respiratory failure and patients with sepsis. In the presence of septic shock, a persistent decrease in functional vessel density (FVD) indicates poor survival when assessing with HVM (82, 83). Normal values for the adult population describe an MFI below 2.6 to differentiate normal from abnormal microcirculation (84). The extrapolation of these values to the pediatric population has yet to be validated.

Microcirculation monitoring could help assess fluid responsiveness. A report done in the adult population by Pranskunas et al. found that patients with clinical signs of impaired organ perfusion and an MFI less than 2.6 were fluid-responsive when MFI increased from 2.3 to 2.5, while patients with an MFI at 2.8 did not respond to fluid resuscitation indicating nonresponsiveness (85). The idea of incorporating microcirculatory targeted treatment in septic shock resuscitation guidelines was tested by van der Voort et al. (86). This group completed a randomized control pilot study to increase microcirculation variables using nitroglycerin, dopamine, enoximone, and dexamethasone. Although the results did not change end organ recovery compared to the control group, this clinical trial introduced the concept of microcirculatory assessment within treatment guidelines.

Data published for neonates and pediatric patients with respiratory failure and candidates for ECMO have also shown microcirculatory abnormalities. MFI values in respiratory failure are relatively high, and heterogeneity index (HI) values are relatively low (58). In these investigations, the findings did not correlate with mortality. In contrast and related to mortality, in pediatric patients (37) nonsurvivors of sepsis presented with elevated MFI and HI levels that did not decrease over time, contrasting with an initial reduced MFI in survivors. In adult patients with septic shock (83) nonsurvivors of sepsis presented with altered small vessel perfusion that did not improve, unlike the improvement noted in the survivor group over time. These microcirculatory findings were present despite similar hemodynamic and oxygenation parameters between survivors and non-survivors. The use of HVM to assess microcirculation over time can serve as a tool for risk stratification and prognostication and guide earlier intervention in sepsis management. The alterations in microvasculature, coupled with endothelial glycocalyx biomarkers, can lead to different management strategies. The emphasis on precision medicine for sepsis can determine which therapies in the future can work best individually (87).

In a recent data set related to other disease processes, the microcirculation assessment in the adult population with COVID-19 demonstrated a dysfunctional endothelial glycocalyx. Reports of elevated endothelial glycocalyx biomarkers (syndecan 1, chondroitin sulfate) (88) along with reduced heparanase-2, elevated ADAMTS13, and endothelial growth factor (89) led patients to a prothrombotic state. Rovas et al*.* combined the endothelial glycocalyx biomarkers with IDF videomicroscopy to quantify vascular density, red blood cell velocity, and glycocalyx dimensions for moderate-to-severe or critical COVID-19. The software used in this study calculates the dynamic lateral movement of RBCs into the permeable part of the glycocalyx layer expressed as the perfused boundary region (PBR in µm), inversely related to the endothelial glycocalyx dimension. The COVID-19 epidemic resulted in a clinical entity in the pediatric population termed multisystem inflammatory syndrome in children (MIS-C), a rare SARS CoV-2 virus infection complication. Fernandez et al. described two children with MIS-C where they investigated the endothelial function by combining videomicroscopy (microcirculation) and the biomarker Syndecan-1 to evaluate the endothelial Glycocalyx (48). In this report, the authors noted endothelial glycocalyx damage in a critically ill child with MIS-C. Degradation of the EG weakens the protection barrier covering the endothelial cells, favoring interstitial edema, capillary leak, fluid retention, and multiple organ dysfunction, progressing to failure with worsening clinical outcomes.

The technology to assess microcirculation continues to evolve with time. The recent investigations combining different strategies to evaluate the microcirculation, the endothelium, and EG suggest the potential benefit of collecting this information in critically ill patients. The routine use of videomicroscopy might provide the platform to assess therapeutic approaches for the resuscitation of the hemodynamically unstable pediatric patient. The data collected in the adult population with septic shock has led to the recognition of specific microcirculatory abnormalities these patients have and the resultant changes noted after therapeutic interventions (15). Despite these findings in microcirculation dysfunction, the information has yet to translate to clinical applicability for diagnoses or treatment assessments. Several studies have demonstrated that the lack of response of the microcirculation to resuscitation is associated with poor outcomes. A potential improvement in noninvasively assessing the microcirculation involves adding endothelial glycocalyx (EG) information. A significant number of biomarkers are available to evaluate both the quality of the glycocalyx and the vessel integrity to fluid extravasation.

The growth in physiological and pathophysiological knowledge has created potential treatment modalities to improve microcirculation. Clinical benefits have been observed in animal shock models where microcirculation and perfusion parameters (red blood cell velocity, functional capillary density) improve with the use of endothelial barrier modulators such as an angiopoietin-1 mimetic vasculotide or platelet-derived growth factor (16). A follow-up review from this group examined the current investigative therapies, including sex hormones and steroid use, proposed to prevent microvascular leakage related to endothelial glycocalyx damage targeting angiopoietin/Tie2 and sphingosine-1 phosphate signaling (90). Potential targeted therapies addressed in these models add understanding and novelty to future therapeutic interventions for critically ill patients.

## Microcirculation and endothelial glycocalyx in pediatrics

Pediatric-specific work has been published indicating age-related developmental changes in the microcirculation (68) and the loss of hemodynamic coherence despite adequate macrocirculatory resuscitation (66) in the critically ill neonate. Despite advances in videomicroscopy, introducing these devices into the pediatric practice has yet to be incorporated. Technical shortcomings, inter-observer, and intra-observer variabilities challenge image analysis. Further, image analysis can be time-consuming (65). Future solutions to ease the assessment of microcirculation into clinical practice include the advancement in machine learning with the inclusion of software with appropriate algorithms allowing for instant analysis. Collecting quality information with the development of new algorithms can improve data processing, along with determining additional functional parameters of microvascular blood flow. Point-of-care assessment requires the development of integrated automated analysis software. There is a need to develop average values reference targeting different age groups. Ideally, integrating and evaluating pediatric clinical outcomes to microcirculatory guided therapies can add to the current hemodynamic assessments in critical care and anesthesiology practices.

Moreover, an essential role of the endothelium, particularly the endothelial glycocalyx, has emerged as having a central role in critical illness (71), and elevation of EG biomarkers such as Syndecan-1, heparan sulfate, hyaluronan can be related to EG-damage patient outcome, particularly with sepsis (91). In a recent review by Richter et al., the authors highlight the limited literature available describing EG pathophysiology in pediatric critical illness compared to adult data and analyze the current state of knowledge related to EG changes in age-maturation and EG alterations during pediatric acute critical illness (34). Fernandez et al. published a systematic review addressing EG alterations in sepsis assessed by biomarkers (92), including mostly adult and a few pediatric patients. The authors’ aimed to determine mortality risk as the primary outcome and respiratory failure and MODS as secondary outcomes. Inclusion criteria required patients with sepsis and abnormal biomarkers indicating glycocalyx injury, as determined by elevated glycocalyx biomarkers (Syndecan-1 and endocan) and clinical outcome descriptions. Their conclusions found a correlation between an abnormal result in biomarker levels and increased risk of death, respiratory failure, and MODS. These results raise the need to understand further the endothelial system's role in health and disease processes. The use of biomarkers in the critically ill pediatric and neonatal population remains an area requiring further investigation.

**Figure 3:**
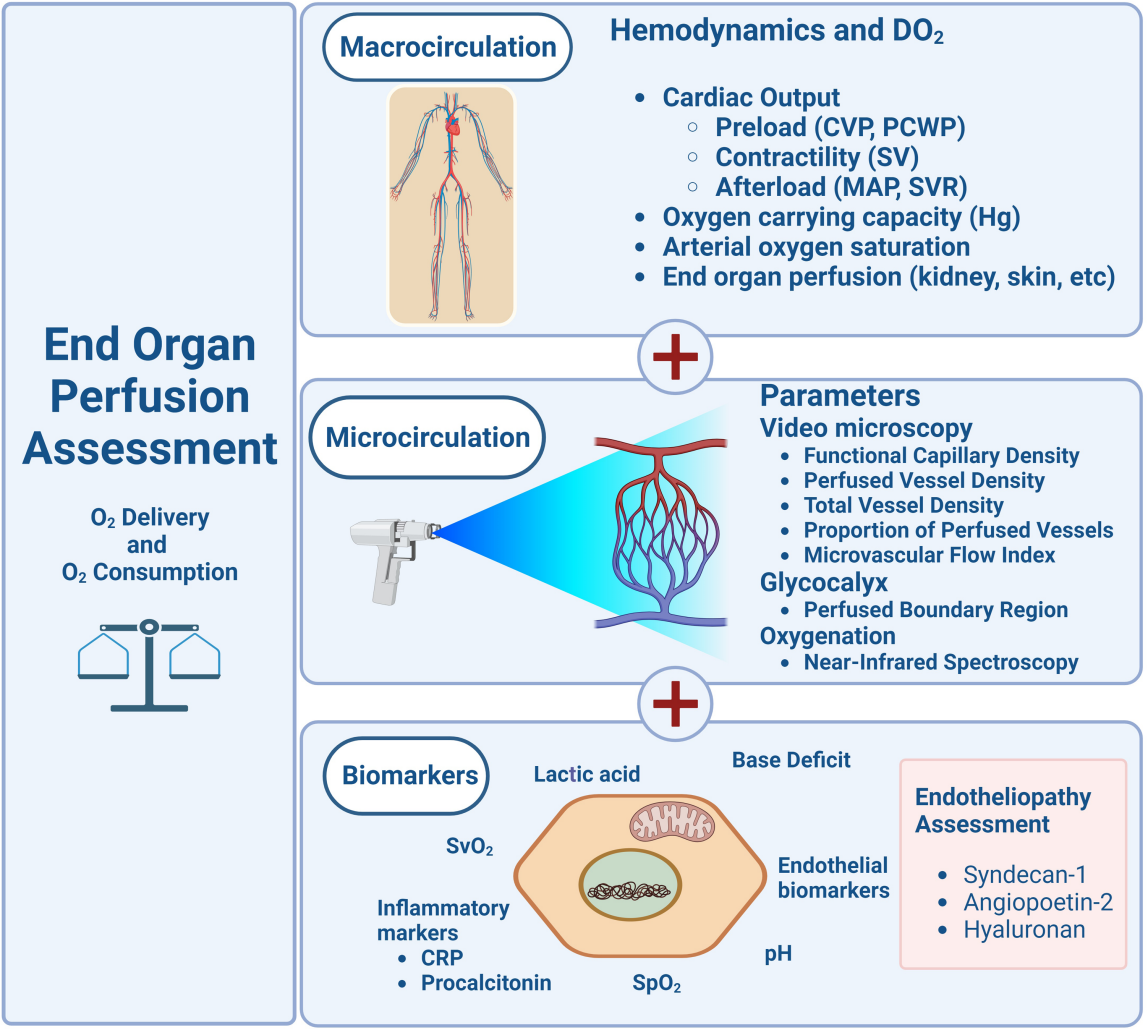
Schematic diagram proposing the evaluation of macro and micro circulations in the pediatric clinical assessment.

The microcirculatory alterations noted in the adult population have led investigators to attempt to combine videomicroscopy with biochemical elements that can provide added information on endothelial glycocalyx dysfunction. The MicroRESUS study (93), a prospective clinical trial to commence in 2023, including adult patients, will examine microcirculatory and mitochondrial function in human patients with circulatory shock undergoing cardiac bypass. The use of specific endothelial glycocalyx biomarkers, as described by Fernandez et al. (94) and Krispinsy et al. (95) in neonates, has opened the opportunity to understand microcirculatory abnormalities and endotheliopathy, escalating our understanding of the impact of critical illness at the end-organ perfusion. With our current state of knowledge, the use of biomarkers, and the technology available, the endothelial glycocalyx can become a treatment-targeted organ in managing critically ill patients. Future medical decision-making and prognostication algorithms can support clinical decision-making for children and neonates (Figure 3) by including microcirculation and endothelium glycocalyx assessments. Understanding epitheliopathy in critical illness and its impact on microcirculation and endothelial glycocalyx function holds promise for new therapeutic approaches to protect and repair the EG. The current understanding of the endothelial system's function in health and disease and its association with clinical outcomes is an ongoing area of investigation.
